# Ferroptosis in intracerebral hemorrhage: a bibliometric overview of mechanisms and future directions

**DOI:** 10.3389/fncel.2026.1813951

**Published:** 2026-06-16

**Authors:** Hanbing Yang, Xuankai Cui, Yilin Liu, Zhipeng Guo, Shuhan Yang, Jingni Wang, Peilin Zhu, Xingchun Wang, Xinrui Wang, Xingxia Wang

**Affiliations:** 1 The People’s Hospital of Renshou County, Renshou, Sichuan, China; 2 Department of Neurology, The Affiliated Hospital, Southwest Medical University, Luzhou, Sichuan, China; 3 Department of Clinical Medicine, School of Clinical Medicine, Southwest Medical University, Luzhou, Sichuan, China; 4 The Affiliated Stomatological Hospital, Southwest Medical University, Luzhou, Sichuan, China

**Keywords:** bibliometrics, ferroptosis, intracerebral hemorrhage, molecular mechanisms, research fronts

## Abstract

**Introduction:**

Research on ferroptosis in intracerebral hemorrhage (ICH) has expanded rapidly in recent years, but the overall knowledge structure and research trends of this field remain unclear.

**Methods:**

A total of 254 publications related to ferroptosis in ICH from the Web of Science Core Collection and Scopus databases (2014–2025) were analyzed using Bibliometrix, VOSviewer, and CiteSpace. Bibliometric analyses were performed to evaluate publication trends, research hotspots, collaboration networks, and emerging themes.

**Results:**

Publication output increased markedly after 2020, reflecting growing attention to ferroptosis-related brain injury after ICH. China contributed nearly 80% of the publications, although international collaboration remained relatively limited. Keyword evolution and co-citation analyses showed that the research focus gradually shifted from general cell death pathways toward more specific mechanisms involving iron metabolism, lipid peroxidation, GPX4-mediated antioxidant regulation, and neuroinflammation. Several highly cited studies published after 2017 played important roles in shaping the development of this field. Recent studies have increasingly focused on downstream pathological processes and potential therapeutic strategies.

**Discussion:**

This study summarizes the major research themes and evolving directions of ferroptosis research in ICH and provides a useful reference for future mechanistic and translational studies.

## 1 Introduction

Stroke can be divided into two major categories, including hemorrhagic stroke and ischemic stroke. The hemorrhagic type primarily includes intracerebral hemorrhage (ICH) and subarachnoid hemorrhage (Pan et al., 2023; Zheng et al., 2025). Clinically, ICH is marked by a sudden onset, often rapid neurological deterioration, leading to disproportionately high long-term mortality and disability rates (Keep et al., 2012). Therefore, ICH is considered one of the most devastating forms of cerebrovascular disease. Although it accounts for only 28.8% (range 28.3–28.8%) of all stroke cases, ICH is responsible for 49.6% (range 49.3–49.8%) of disability-adjusted life-year counts related to stroke (GBD 2021 Stroke Risk Factor Collaborators, 2024; Feigin et al., 2025). This creates a major burden on public health (GBD 2021 Diseases and Injuries Collaborators, 2024). Established modifiable risk factors include hypertension, diabetes mellitus, tobacco use, dyslipidemia, and systemic infection (Chutinet et al., 2025; Lee, 2025). Nonmodifiable risk factors include advanced age, male sex, Asian ethnicity, cerebral amyloid angiopathy, cerebral microbleeds, and chronic kidney disease. Among these, hypertension is a key risk factor for ICH. This is especially true for cases with persistently high blood pressure. It is regarded as the strongest and most common factor in this category (Lee, 2025). Recently, clinical management of ICH remains limited. Clinical care focuses on three main areas: symptom management, blood pressure control, and surgical removal of hematomas (Pradilla et al., 2024). However, it remains unclear whether these approaches actually improve long-term neurological function in patients. Many neuroprotective agents succeeded in preclinical studies but later failed in clinical trials. Despite some progress, treatment of ICH still faces major challenges. Drugs often cannot cross the blood–brain barrier effectively. They may also break down too quickly in the body or lack precise targeting (Pan et al., 2023). Therefore, understanding the molecular basis of brain injury after ICH is now a critical goal. Finding new and effective treatment targets has become equally urgent.

After ICH, brain damage does not occur instantly but develops progressively in stages. The first hit comes mainly from the clot itself; from its sheer size and the physical pressure it puts on the brain. Unlike primary injury, secondary injury following ICH encompasses a more intricate pathophysiological cascade. It is characterized by mitochondrial dysfunction, robust efflux of neurotoxic substances, and extensive release of inflammatory mediators (Pan et al., 2023). A key mechanism underlying neuronal loss and neurological deficits is the activation of programmed cell death pathways. In addition to established forms such as apoptosis, necroptosis, and autophagy, ferroptosis has attracted growing attention. First identified in 2012, ferroptosis is a non-apoptotic, iron-dependent mode of cell death driven by lipid peroxidation. Because of the pronounced roles of iron overload and oxidative stress after hemorrhage, ferroptosis is considered highly relevant to brain damage after ICH (Dixon et al., 2012).

In recent years, this research field is expanding rapidly, yet studies remain fragmented. Different groups focus on distinct molecular targets within the ferroptosis pathway, including GPX4 and ACSL4 (Stockwell et al., 2017), and adopt diverse models and outcome measures. A systematic integration of the overall research landscape, collaboration networks, and evolutionary trends is absent. Traditional narrative reviews are not equipped to objectively or quantitatively delineate the knowledge structure, research frontiers, or future trajectories of the field (Hoang, 2025). Bibliometrics, a quantitative analytical approach grounded in mathematics and statistics, enables the synthesis of dispersed findings, identification of high-impact publications, core authors and institutions, and cross-country collaborative patterns. It also captures the evolution of research hotspots via keyword co-occurrence analysis (Hicks et al., 2015; Tan et al., 2024). Accordingly, this study applies bibliometric methods to systematically examine the literature on ferroptosis in ICH. This study aims to present publication growth trends and the distribution of core research forces, to identify major research themes and their dynamic evolution, and to predict potential research frontiers and knowledge gaps. Through this analysis, we aim to provide a clear intellectual map for future investigators and facilitate the clinical translation of ferroptosis-based interventions for ICH (Ninkov et al., 2022).

## 2 Methods and materials

### 2.1 Data sources and search strategy

A systematic literature search was conducted on January 19, 2026, using the Web of Science Core Collection (WoSCC) (Öztürk et al., 2024) and Scopus (Burnham, 2006) to identify publications on ferroptosis in ICH. The WoSCC search strategy was as follows: TS = (ferroptosis OR “iron-dependent cell death”) AND TS = (“cerebral hemorrhage” OR “intracerebral hemorrhage” OR ICH). The Scopus search strategy was as follows: (TITLE-ABS-KEY (ferroptosis OR “iron-dependent cell death”)) AND (TITLE-ABS-KEY (“cerebral hemorrhage” OR “intracerebral hemorrhage” OR ICH)). The search time span was set from January 1, 2014, to December 31, 2025, to ensure comprehensive coverage of ferroptosis studies related to ICH. The search scope was restricted to “articles” and “reviews” published in English in both databases. A total of 417 records were initially identified, with 218 from WoSCC and 199 from Scopus.

Title and abstract screening were performed by a single reviewer, as consistency in the initial assessment was ensured by predefined criteria for document type and topic relevance. Any uncertainties were resolved through discussion among all authors.

### 2.2 Data extraction and processing

**Figure 1:**
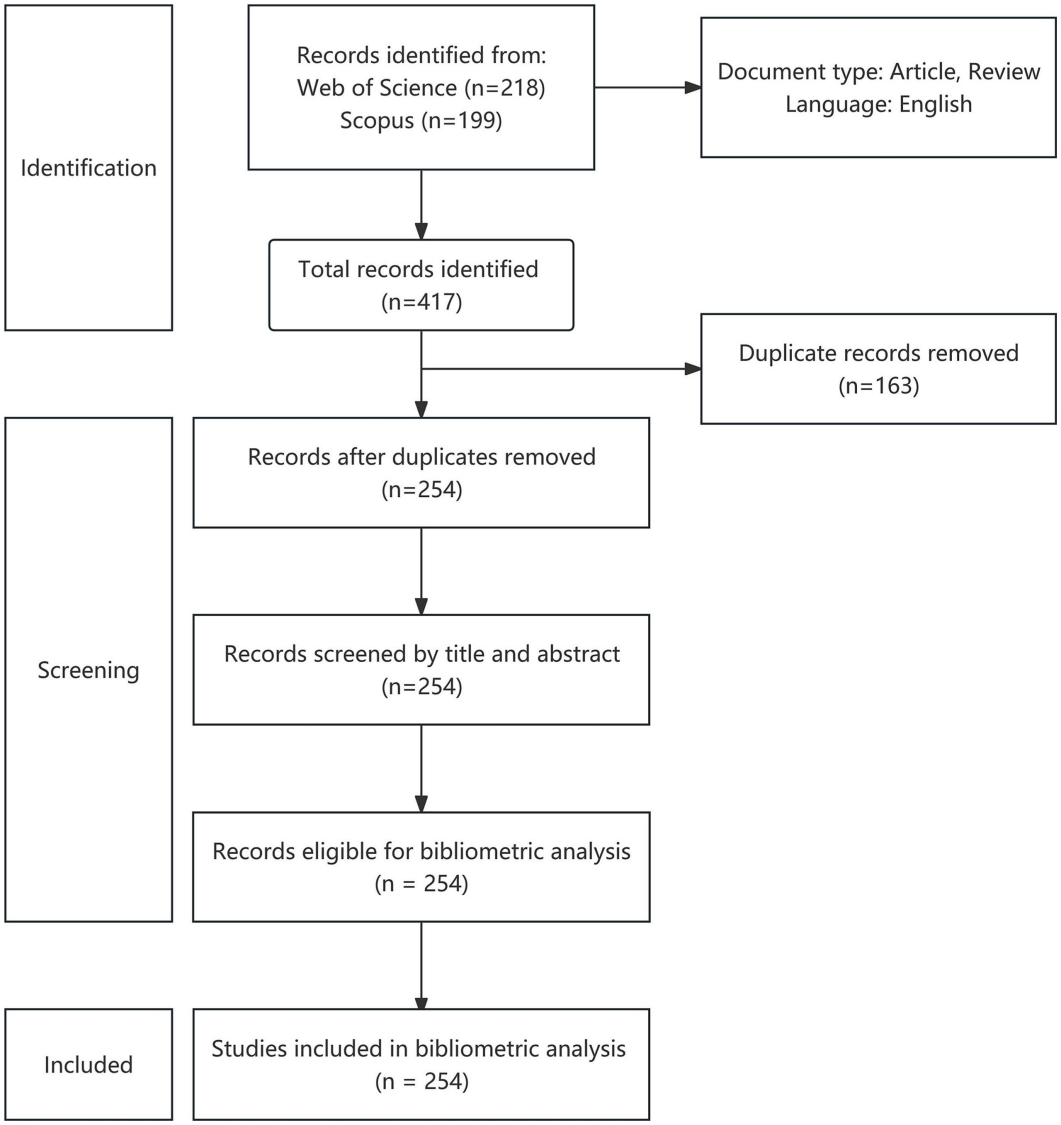
Flowchart of study identification, screening, and inclusion.

Records exported from WoSCC and Scopus were retained in their original formats (plaintext and BibTeX, respectively). Bibliographic data were imported into R software (version 4.4.1) and processed using the bibliometrix package (version 5.2.1). Records from Scopus were converted to BibTeX format using the *convert2df()* function, while records from WoSCC were converted to ISI plaintext format. Datasets were merged with the *mergeDbSources()* function, and duplicates were automatically identified and removed based on DOI and title matching. After removing 163 duplicates, 254 unique publications were retained for bibliometric analysis. The detailed filtering process was illustrated in Figure 1.

Both original research articles and review articles were included in this analysis, as review articles contribute to shaping the intellectual structure and tracing the thematic development of the research field. Among the 254 publications included in this study, 165 were original research articles and 89 were review articles.

### 2.3 Bibliometric analysis and visualization

Data organization and preliminary analysis were conducted in Microsoft Excel 2021 (Hao et al., 2025; Divisi et al., 2017). Bibliometric analysis utilized various data visualization and analysis tools (Yan et al., 2025), including the bibliometrix R package and its web interface Biblioshiny (Aria and Cuccurullo, 2017), VOSviewer (version 1.6.10) (van Eck and Waltman, 2017), and CiteSpace (6.4.R1) (Chen, 2004).

Bibliometrix is an open-source bibliometric analysis package based on the R language that supports a complete workflow from data retrieval and cleaning to analysis and visualization (Arruda et al., 2022). In this study, Bibliometrix was applied to generate a multi-faceted analysis, which included publication outputs categorized by country and author, identification of core journals, keyword frequency and trend mapping, as well as detection of influential references. Visualizations were created via the integrated Biblioshiny interface after data processing.

VOSviewer is a Java-based free software by Van Eck and Waltman in 2009, which is designed for processing large-scale data and specializes in the visualization of bibliometric networks (Hu et al., 2024). The full counting method was applied in VOSviewer to generate co-authorship networks by country and keyword co-occurrence networks (Yan et al., 2024). In the network diagrams, node size reflected the number of articles or co-occurrence frequency; the different colors of nodes represented different categories of research topics; links between nodes indicated the co-occurrence relationship, while link width represented the co-occurrence frequency between two nodes (Chen et al., 2023; Wu et al., 2022). Cluster analysis was conducted using the default VOS algorithm in VOSviewer (Xie et al., 2025). A concept was included in the network if it appeared or was cited at least five times; thresholds were adjusted to enhance network clarity. Link strengths were calculated using the association strength normalization method, and the network layout was optimized by tuning the attraction and repulsion parameters.

CiteSpace was used to detect bursts in keywords and reference citations through Kleinberg’s burst detection algorithm to highlight emerging trends and shifts within research hotspots (Zhang R. et al., 2025; Wang et al., 2024). Parameter settings were as follows: time slicing from 2014 to 2025 (1 year per slice), node type depending on analysis objective (keyword or reference). The “Pathfinder” method was used for path selection, while all other parameters were set to their default values (Jiang et al., 2025).

In the frequency-based keyword analysis, terms such as “ferroptosis” and “intracerebral hemorrhage” were excluded to emphasize mechanistic themes in the literature. Conversely, these terms were retained in the keyword co-occurrence network analysis to maintain the conceptual framework and illustrate associations with key mechanistic pathways.

## 3 Results

### 3.1 Annual publication trends

**Figure 2:**
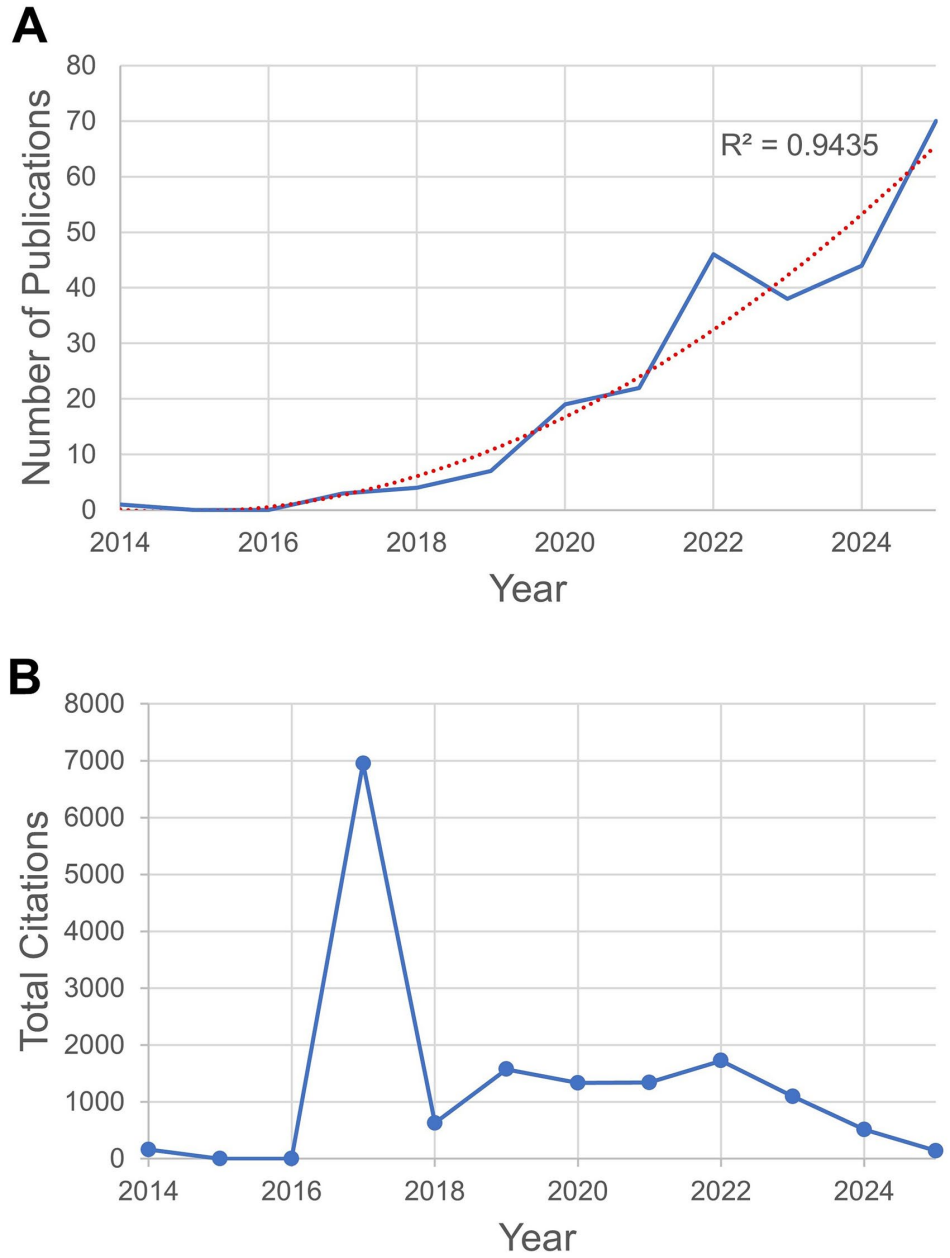
Annual publication trends and citation impact in ferroptosis and ICH research (2014–2025). **(A)** Annual publication output and regression fitting analysis. **(B)** Total citation counts by publication year.

Research on ferroptosis in ICH demonstrated a clear upward trend from 2014 to 2025 (Figure 2A). After a relatively stable period between 2014 and 2019, publication output increased markedly from 2020 onwards, indicating a phase of rapid expansion.

To further characterize this trend, both linear and nonlinear regression models were evaluated. Linear regression showed a significant increasing trend (*R*^2^ = 0.8526, *p* < 0.001). In comparison, nonlinear models provided a better fit, with a quadratic model showing a higher *R*^2^ value (*R*^2^ = 0.9435). The third-order model showed only minimal improvement (*R*^2^ = 0.9443). The publication trend was better described as a nonlinear and accelerated growth process.

To further assess research impact over time, total citations by publication year were analyzed (Figure 2B). Citation counts increased markedly after 2017, indicating the emergence of highly influential studies during this period. Citation levels remained relatively high between 2019 and 2022, reflecting sustained academic attention to ferroptosis in ICH. In contrast, citation counts declined in recent years (2023–2025), likely due to the shorter time available for newly published articles to accumulate citations.

### 3.2 Contributions of countries and regions

**Figure 3:**
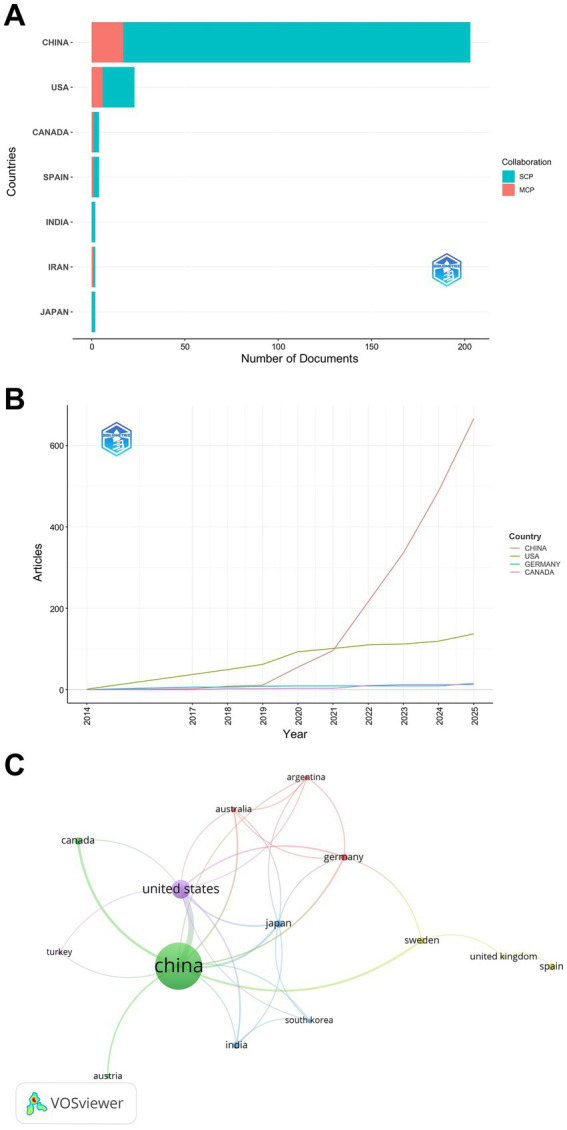
Geographical and collaborative landscape of publications: **(A)** Distribution of corresponding authors’ countries and collaboration patterns; **(B)** Temporal trends in national research participation based on author affiliations; **(C)** International collaboration network.

To assess the contribution of different countries and regions, a geographical analysis was conducted. China was the dominant contributor with 203 articles (79.9% of the total) substantially surpassing the United States (23 articles, 9.1%) and other nations (Figure 3A). Table 1 presents the main contributing countries based on corresponding author analysis. Temporal analysis showed that research participation from China increased markedly after 2020, whereas the growth trends in the United States and other countries were comparatively less pronounced (Figure 3B).

**Table 1:** Top 7 most relevant countries by corresponding author.

Country	Articles	Articles %	SCP	MCP	MCP %
China	203	79.9	186	17	8.4
USA	23	9.1	17	6	26.1
Canada	4	1.6	3	1	25
Spain	4	1.6	3	1	25
India	2	0.8	2	0	0
Iran	2	0.8	1	1	50
Japan	2	0.8	2	0	0

The analysis of international collaboration showed that Single Country Publications (SCP) were the most common. Among countries with relatively high publication outputs, the United States had a higher rate of collaboration with 26.1% of its publications being Multiple Country Publications (MCP), compared to 8.4% for China (Figure 3A; Table 1). For countries with low publication counts, MCP percentages should be interpreted with caution due to limited sample size.

The collaborative network (Figure 3C) showed the core relationship of China and the United States. Moreover, the network was organized into separate clusters, where China and the United States were the main hubs of their respective partners.

### 3.3 Impact of journal distribution and sources

**Figure 4:**
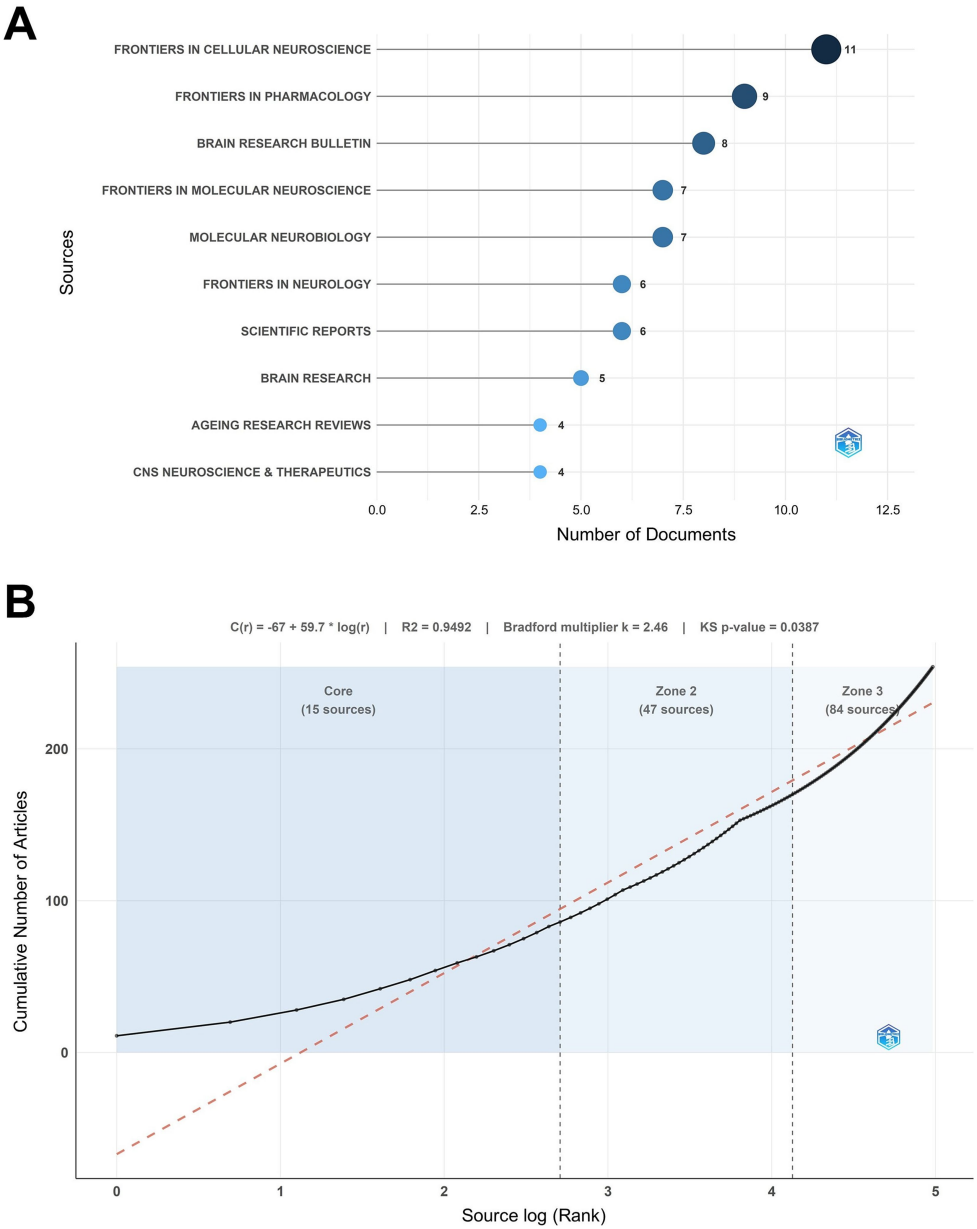
Core journals and source impact. **(A)** Top 10 most prolific journals. **(B)** Bradford’s law analysis identifying core sources.

Journal distribution was studied to determine the fundamental knowledge in the study area of ferroptosis in ICH. In Figure 4A, the top ten journals were identified with the top one being Frontiers in Cellular Neuroscience (11 publications), then Frontiers in Pharmacology (9 publications), and then Brain Research Bulletin (8 publications). The inclusion of journals such as Molecular Neurobiology and Frontiers in Molecular Neuroscience underscores the field’s foundation in molecular mechanisms.

Bradford’s Law was further applied to evaluate the distribution of journals in this field (Figure 4B). The journals were divided into three productivity zones, including a core zone containing 15 journals, followed by zone 2 with 47 journals and zone 3 with 84 journals. Most of the highly productive journals shown in Figure 4A were located within the Bradford core zone, indicating their major role in this research area. A relatively small number of journals contributed a large proportion of publications, whereas the remaining studies were distributed across a broader range of peripheral journals. In addition, the fitted Bradford curve showed good agreement with the observed distribution (R^2^ = 0.9492).

### 3.4 Keyword analysis

**Figure 5:**
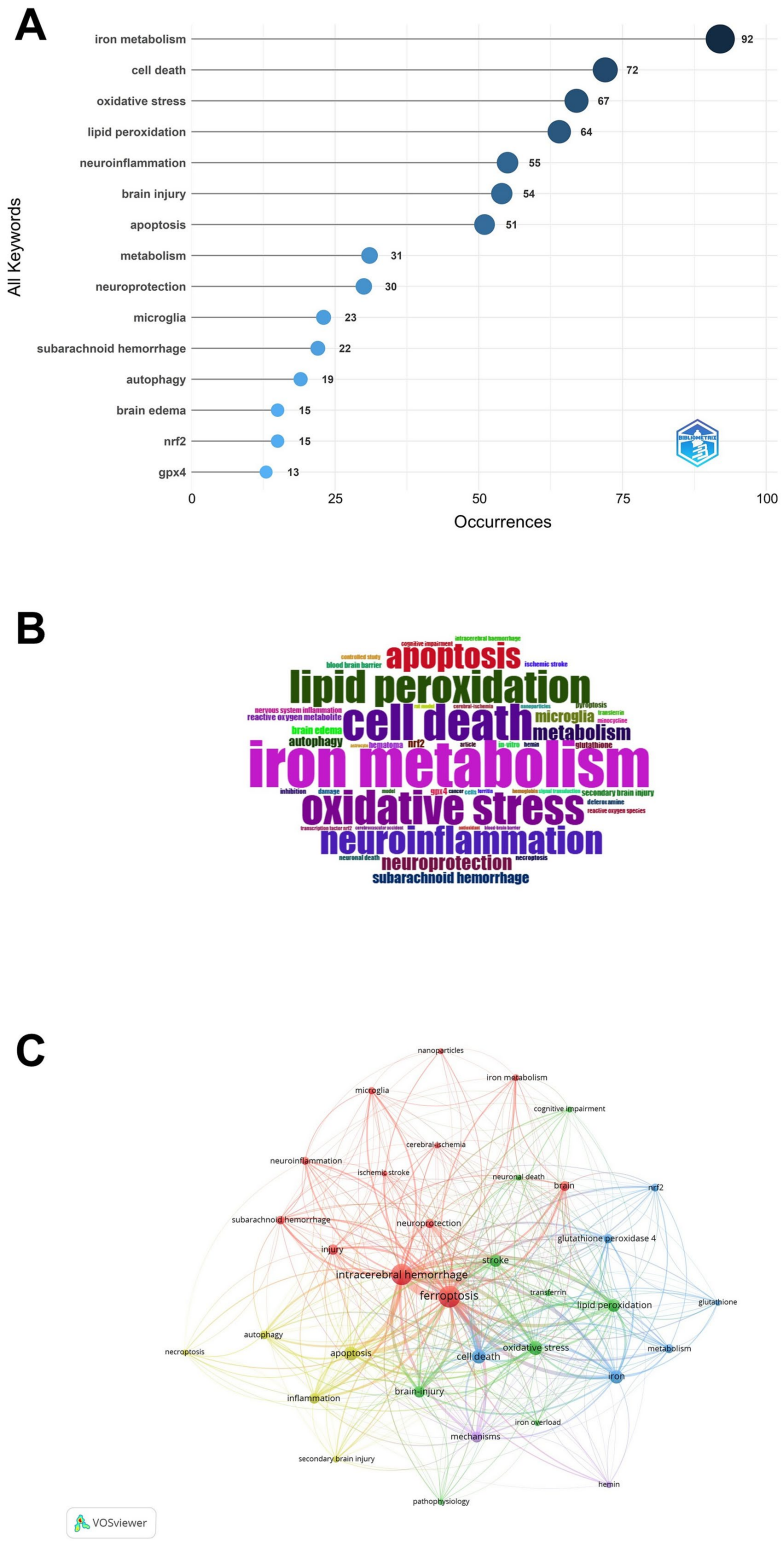
Keyword analysis and thematic clusters. **(A)** Top 15 high-frequency keywords. **(B)** Word cloud of research foci. **(C)** Co-occurrence network of keywords clustered by thematic areas.

Keywords play a core role in an article, and by analyzing keywords, we can explore hotspots in a research field (Wang et al., 2024). In this study, keywords were extracted from all publications and analyzed using VOSviewer and Bibliometrix (Wu et al., 2021). Figure 5A listed the 15 most frequent keywords. “Iron metabolism” ranked first with an occurrence frequency of 92, significantly higher than other terms. Other high-frequency keywords (occurrences > 50) were “cell death” (72 times), “oxidative stress” (67 times), “lipid peroxidation” (64 times), “neuroinflammation” (55 times), “brain injury” (54 times), and “apoptosis” (51 times). To visually represent the research landscape, high-frequency keywords from the literature were compiled into a word cloud, as shown in Figure 5B. The size of each keyword was proportional to its frequency of occurrence, highlighting core research directions. The word cloud revealed that “iron metabolism,” “cell death” and “oxidative stress” were most high-frequency keywords, reflecting core research themes. Other prominent terms included “lipid peroxidation,” “neuroinflammation” and “apoptosis.”

The keywords with a frequency of occurrence greater than 7 times were extracted and clustered by VOSviewer (Feng et al., 2025). The nodes’ different colors represented the five clusters found in Figure 5C. (1) Cluster1 (red), which contained 12 keywords, primarily concerned the core intersection between cerebrovascular diseases and ferroptosis. Keywords in this cluster included ferroptosis, intracerebral hemorrhage and cerebral-ischemia. (2) Cluster2 (green), which contained 9 keywords, was mainly correlated with the pathophysiology and molecular mechanisms of brain injury. Keywords in this cluster included lipid peroxidation, iron overload and brain-injury. (3) Cluster3 (blue), which contained 6 keywords, associated with the core defense system of cellular antioxidant defense. Keywords in this cluster included iron, cell death and glutathione peroxidase 4. (4) Cluster4 (yellow), which contained 5 keywords, concentrated on programmed cell death patterns and secondary injury. Keywords in this cluster included apoptosis, autophagy and inflammation. (5) Cluster5 (purple), which contained 2 keywords, focused on exploration of the mechanism of the specific model. Keywords in this cluster included hemin and mechanisms (Feng et al., 2025; Li et al., 2024; Zhang M. et al., 2025). These clusters formed an integrated network, where ferroptosis and intracerebral hemorrhage functioned as connecting hubs, while lipid peroxidation and multiple cell death pathways acted as convergent mechanisms in disease progression.

### 3.5 Research evolution and trends of research

**Figure 6:**
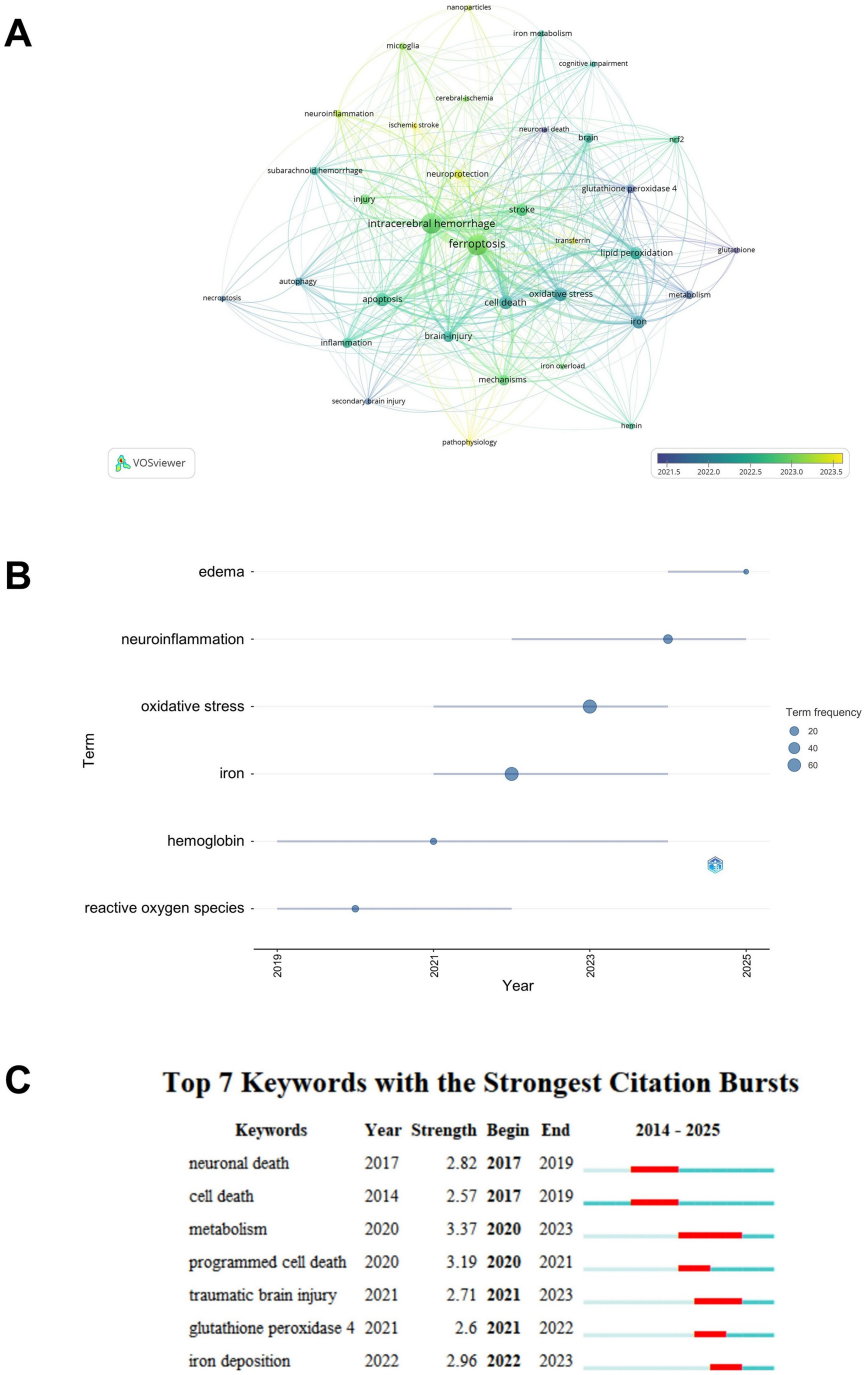
Temporal evolution of research themes and fronts. **(A)** Overlay visualization of keyword co-occurrence over time. **(B)** Trend topic analysis. **(C)** Top keywords with the strongest citation bursts.

The overlay visualization of keyword co-occurrence networks depicted how research themes evolved over time (Figure 6A). Themes in purple (predominantly pre-2022), such as “necroptosis” and “secondary brain injury,” represent foundational pathological concepts. Themes in green (around 2022), such as “apoptosis,” “autophagy” and “lipid peroxidation” signal a shift toward investigating specific mechanisms. The most recent themes, in yellow-green (2023 onward), such as “microglia” and “neuroprotection,” highlight the current expansion into detailed cellular mechanisms and long-term functional outcomes.

This temporal change was quantified by trend topic analysis (Figure 6B). Early keyword terms were “reactive oxygen species” (median year 2020) and “hemoglobin” (median 2021), and then a spurt in attention towards core mechanism terms such as “iron” (median 2022) and “oxidative stress” (median 2023). The latest themes include “neuroinflammation” (median 2024) and “edema” (median 2025), which validated the direction the field is currently taking, which is downstream pathological outcomes.

Further division of the research fronts was made by analyzing keywords having the highest citation bursts (Figure 6C). The first waves (2017–2019) were set at the level of broad conceptual terms (such as “neuronal death” and “cell death”), and then there was a time of intensive research (2020–2022) on concrete mechanisms (“metabolism,” “programmed cell death,” “glutathione peroxidase 4”). The latest bursts include specific interest in “traumatic brain injury” (2021–2023) and “iron deposition” (2022–2023) which suggests a trend toward comparative studies across related diseases and more detailed analysis of pathological features.

### 3.6 Knowledge base and research fronts

The intellectual foundation and development of the field were outlined by analyzing the most globally referenced documents and references that had high citation bursts.

**Figure 7:**
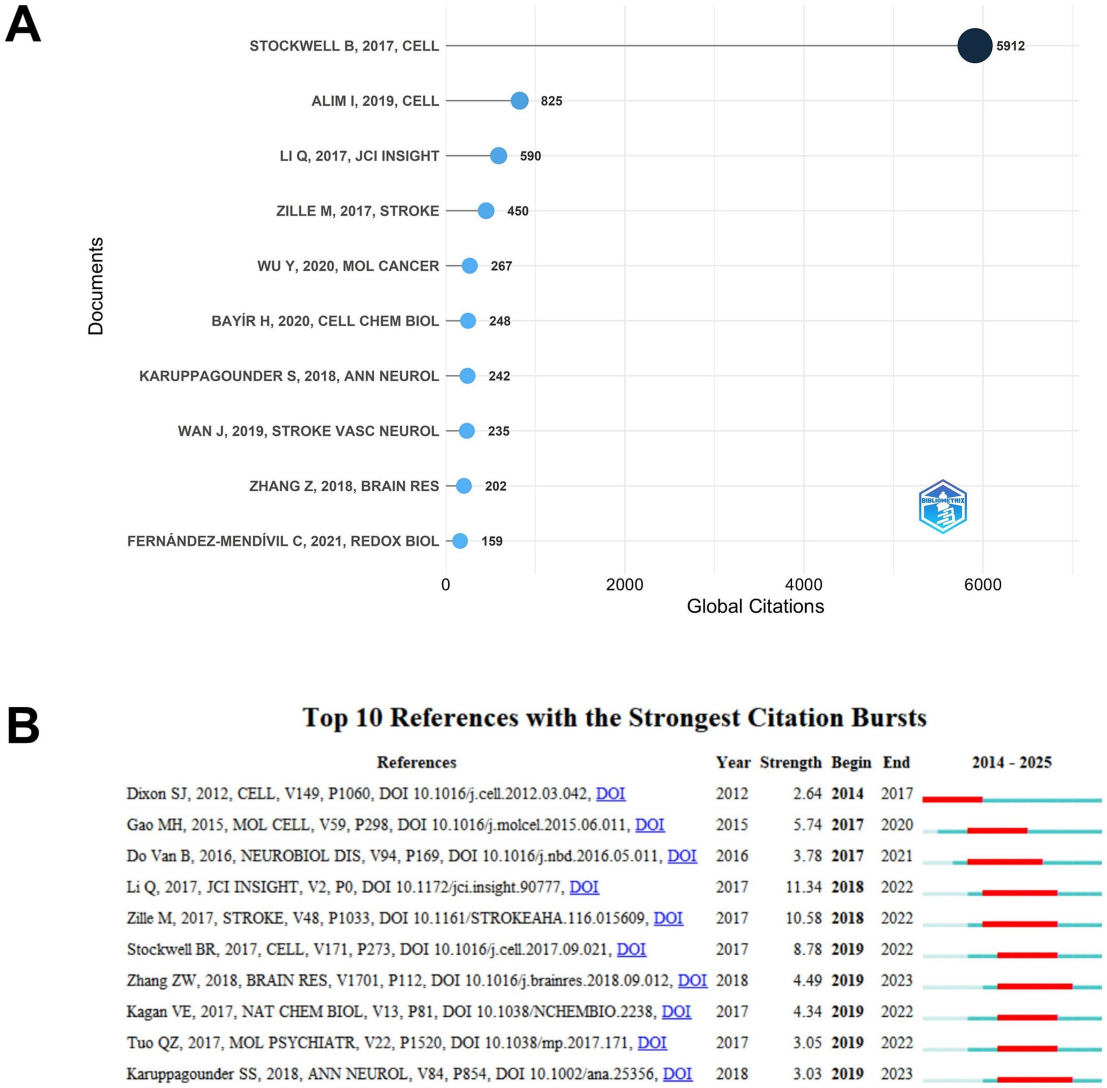
Intellectual base and dynamic research fronts. **(A)** Most globally cited documents. **(B)** References with the strongest citation bursts.

The core knowledge base was identified in the course of the analysis of the most cited documents (Figure 7A; Table 2). Stockwell et al. (2017) published a landmark review by Cell and quickly became the influential background publication, having 5,912 citations worldwide (Stockwell et al., 2017). The important articles such as those by Alim et al. (2019), Zille et al. (2017), and Li et al. (2017) constitute the main body of knowledge on ferroptosis as applied to ICH (Alim et al., 2019; Li et al., 2017; Zille et al., 2017). The highest normalized citation score (3.82) was given to the review by who brings up the facts concerning cancer progression but explicitly mentions ICH. This shows that powerful mechanistic knowledge in related disciplines form significant aspects in the basic knowledge of the field.

**Table 2:** Most global cited documents.

First author (year)	Article title	Journal	Total citations	Normalized TC
Stockwell et al. (2017)	Ferroptosis: a regulated cell death nexus linking metabolism, redox biology, and disease	Cell	5,912	2.55
Alim et al. (2019)	Selenium drives a transcriptional adaptive program to block ferroptosis and treat stroke	Cell	825	3.67
Li et al. (2017)	Inhibition of neuronal ferroptosis protects hemorrhagic brain	JCI Insight	590	0.25
Zille et al. (2017)	Neuronal death after hemorrhagic stroke *in vitro* and *in vivo* shares features of ferroptosis and necroptosis	Stroke	450	0.19
Wu et al. (2020)	The epigenetic regulators and metabolic changes in ferroptosis-associated cancer progression	Molecular Cancer	267	3.82
Bayır et al. (2020)	Achieving life through death: redox biology of lipid peroxidation in ferroptosis	Cell Chemical Biology	248	3.55
Karuppagounder et al. (2018)	N-acetylcysteine targets 5 lipoxygenase-derived, toxic lipids and can synergize with prostaglandin E2 to inhibit ferroptosis and improve outcomes following hemorrhagic stroke in mice	Annals of Neurology	242	1.55
Wan et al. (2019)	Iron toxicity, lipid peroxidation and ferroptosis after intracerebral haemorrhage	Stroke and Vascular Neurology	235	1.05
Zhang et al. (2018)	Glutathione peroxidase 4 participates in secondary brain injury through mediating ferroptosis in a rat model of intracerebral hemorrhage	Brain Research	202	1.29
Fernández-Mendívil et al. (2021)	Protective role of microglial HO-1 blockade in aging: Implication of iron metabolism	Redox Biology	159	2.61

Citation burst analysis identified the active research fronts (Figure 7B). According to the reference by Li et al. (2017) (Strength = 11.34) and Zille et al. (2017) (Strength = 10.58), there was the citation burst in 2018–2022 (Li et al., 2017; Zille et al., 2017). The burst activity was seen to cluster specifically, six out of the top ten references had the highest bursts published around 2017. This powerful group phase coincided with the increased annual publication growth, indicating that these works have become a knowledge base that advances the research front.

## 4 Discussion

Bibliometric analysis indicates that the number of published papers on ferroptosis in ICH from 2014 to 2025 has increased significantly. Most major journals are distributed in fields such as neuroscience, and the clear classification suggests that a basic knowledge system in this area has been established. The research may be roughly divided into three stages. At first, researchers mainly focus on basic pathological concepts such as cell death, laying a foundation for subsequent studies. Then, researchers pay attention to related molecular mechanisms. Research interest in oxidative stress and other themes gradually grows. In recent years, the key words have shifted towards downstream pathological reactions and treatment strategies.

### 4.1 Research hotspots

#### 4.1.1 Iron metabolism

Ranking first in keyword frequency, iron metabolism tends to be a core hotspot in this field. Its popularity peaked around 2022, forming a close co-occurrence network with iron overload and iron deposition. The number of published papers increased dramatically in this period. Many factors may jointly contribute to the formation of this research hotspot. After ICH, red blood cells rupture and release large amounts of hemoglobin. Free iron resulting from decomposition accumulates in brain cells, causing ferroptosis (Sun et al., 2023). The pathological process suggests that iron metabolism serves as a central part in ferroptosis. Core papers might play an important guiding role. Li et al. (2017) took the lead in verifying the presence of ferroptosis in ICH animal models. This paper achieves a citation burst strength of 11.34, which may prompt a surge in studies on iron metabolism (Li et al., 2017). Numerous studies on iron transporters such as transferrin receptor 1 and iron chelators have been published, which may help to form this hotspot (Ren et al., 2022). Some studies suggest that pyridoxal isonicotinoyl hydrazone and B355252 might exert iron-chelating effects, providing more possible options for the intervention of ICH (Zhang et al., 2021; Han et al., 2025).

#### 4.1.2 Lipid peroxidation and GPX4

The keyword frequencies of lipid peroxidation, oxidative stress, and GPX4 rank highly, and the median research heat is concentrated from 2021 to 2023. The three may together form another core research cluster after iron metabolism. Reactive oxygen species can induce peroxidation reactions in polyunsaturated fatty acids, leading to cellular structural damage (Sun et al., 2023). GPX4, a crucial defensive enzyme, can convert lipid peroxides back into harmless lipids (Cao et al., 2023). The balance between the two influences the process of ferroptosis, and its unique function makes this system important. Stockwell et al.’s (2017) paper has gained 5,912 total citations, which could be regarded as a landmark work in the field. It explains the lipid peroxidation process and the function of GPX4 in ferroptosis, which may help maintain sustained research interest. Abundant intervention targets may make this research hotspot stay widely concerned. Studies indicate that *baicalin* might reduce iron deposition in mice brains and increase GPX4 expression (Duan et al., 2021). Other targets like arachidonate 5-lipoxygenase and Nrf2 are also believed to regulate ferroptosis (Karuppagounder et al., 2018).

#### 4.1.3 Multi-pathway regulation and neuroinflammation

Before 2022, researchers mainly focused on apoptosis, autophagy, necroptosis, and other cell death pathways. A well-cited study by Zille et al. (2017) shows that neuronal death after ICH has characteristics of both ferroptosis and necroptosis, which might encourage relevant studies to shift from a single pathway to multiple pathways. Neuroinflammation, microglia and neuroprotection have attracted rising attention since 2023. After ICH, microglia alter the level of iron transporters to induce ferroptosis, and release inflammatory factors like IL-1β to worsen neuroinflammation (Liu et al., 2025). Severe neuroinflammation may speed up cell death, creating a vicious cycle. There exists a close correlation between cell death pathways and neuroinflammation, gradually emerging as a new research hotspot. It is reasonable that this research hotspot emerges. After progress is made in studying upstream molecular mechanisms, researchers begin to focus on downstream pathological outcomes and intervention methods.

### 4.2 Geographical distribution

According to regional analysis, China publishes 203 papers, accounting for nearly 80 percent of global studies, far more than the second-ranked country. This fact puts China in a leading position in this field. Other countries publish fewer than five papers, with limited reference value. Many factors may lead to this research pattern. From epidemiological data, China had about 26.34 million stroke patients in 2021, including 4.39 million cases of hemorrhagic stroke (National Program for Stroke Prevention and Million Disability Reduction Initiative Expert Committee, 2025). The huge patient population, limited treatments and poor prognosis might drive this field to develop continuously. In recent years, the National Natural Science Foundation of China has funded key topics such as ferroptosis and neuroprotection in neuroscience. Sufficient funds may help produce plenty of research results. The advancement of the ferroptosis experimental system also attracts many research groups to engage in related studies. However, we should also objectively view the existing problems in this field. China has a lower proportion of MCP than the United States, and more international cooperation will be required in the future. The emergence of abundant achievements may suggest a possible trend of blind follow-up in domestic academic publishing.

### 4.3 Limitations

Here we introduce a review of current research at the intersection of ICH and ferroptosis based on bibliometric analysis. Needless to say, some limitations of the review exist. For one, we limited our search to English-language articles and reviews in Web of Science and Scopus. Excluding publications outside of English, case reports, and conference abstracts, we might have missed regionally important findings particularly important findings from traditional medicine communities in which English may not be the academic language. It could shift subtly the global representation of emerging ideas. Data processing has a natural uncertainty. Author name disambiguation, and institution name normalization, for instance, are never completely error-free (even with good databases). In addition, review articles generally accumulate citations more rapidly than original studies, which may introduce citation-related bias in analyses of highly cited publications and knowledge structures. Citation-based indicators are also influenced by time, as recently published studies have less opportunity to accumulate citations compared with older publications. That said, Web of Science and Scopus remain two of the most reliable collections of scientific research. Given the coverage of the core journals in this field, we feel that our dataset covers the vast majority of highly cited contributions. These limitations are all there, but they do not compromise the general reliability of our conclusions.

### 4.4 Future directions

Indeed, we have now a thorough knowledge of ferroptosis after ICH. All key pathways and responsible molecules have now been mapped out. Various Inhibitors (Stockwell et al., 2017) like Ferrostatin-1 and deferoxamine have indeed shown neuroprotective effects in the lab. However, to take laboratory discoveries to effective clinical therapies, we cannot only understand the underlying biology. We need to change our approach and rethink from first principles how this work should be done and assessed.

#### 4.4.1 Capturing the spatiotemporal dynamics of ferroptosis after ICH

From the keyword burst analysis (Figure 6C), we can see that glutathione peroxidase 4 and iron deposition are still the current research hotspots. However, the trend theme analysis (Figure 6B) shows that the research focus is gradually shifting to downstream pathological events, while the dynamic process of ferroptosis after ICH has not been explored. In addition, the reference burst analysis (Figure 7B) shows that the six most influential studies so far have mainly focused on static mechanism verification. In order to make up for the knowledge gap identified in Bibliometric mapping, it may be worth trying to establish a closed loop framework of observation modeling intervention by combining longitudinal analysis observation with computational modeling.

#### 4.4.2 Building a clinically grounded research foundation

The current keyword co-occurrence analysis (Figure 5C) shows that most of the existing literatures focus on the molecular mechanism. However, clinical related terms such as comorbidity, geriatric model and delayed intervention are not prominent in the co-occurrence network, which may indicate that there is a certain distance between basic research and clinical practice. Considering that China has contributed 80% of the relevant literatures (Table 1) and faces the challenge of elderly patients with hypertension and diabetes, the feasible direction in the future is to optimize the preclinical trials. Specifically, elderly animals and comorbid animal models can be introduced into the study, and intervention time more in line with clinical practice can be set.

## 5 Conclusion

This bibliometric study explored the research landscape and developmental trends of ferroptosis in ICH from 2014 to 2025. Research output in this field has increased rapidly, particularly since 2020, with growing attention directed toward molecular mechanisms and downstream pathological processes. Keyword evolution and co-citation analyses showed that the research focus has gradually shifted from general cell death pathways to more specific mechanisms involving iron metabolism, lipid peroxidation, GPX4-mediated antioxidant regulation, and neuroinflammation. Although China contributed the majority of publications in this field, international collaboration remains relatively limited.

Overall, this study provides a comprehensive overview of the knowledge structure and research evolution of ferroptosis in ICH. These findings may help researchers identify major research themes and emerging directions, while also providing a reference framework for future basic and translational studies targeting ferroptosis-related brain injury after ICH.

## Data Availability

The datasets analyzed in this study were obtained from the Web of Science Core Collection and Scopus databases. Further inquiries can be directed to the corresponding author.
